# Editorial: New Advances in Diagnostic Tools and Rehabilitation of Disorders of Consciousness in the Acute Phase

**DOI:** 10.3389/fneur.2021.770791

**Published:** 2021-11-16

**Authors:** Alessandro Pincherle, Mario Rosanova, Karin Diserens

**Affiliations:** ^1^Hopitaux Robert Schuman, Luxembourg, Luxembourg; ^2^Acute Neurological Rehabilitation Unit, Department of Clinical Neurosciences, Lausanne University Hospital, Lausanne, Switzerland; ^3^Department of Neurorehabilitation, IRCSS Istituto Clinico Scientifico Maugeri, Milan, Italy; ^4^Department of Biomedical Sciences and Clinics Luigi Sacco, Faculty of Medicine and Surgery, University of Milan, Milan, Italy

**Keywords:** disorders of consciousness, EEG, evoked potential, cognitive motor dissociation, coma, TMS, TCDS

Disorders of consciousness(DOC) occur in nearly 20% of patients who underwent a brain injury due to different etiologies (1). Accurately assessing the presence of consciousness in DOC patients is crucial to render a reliable prognosis and hence leading to the most appropriate therapeutic and rehabilitative decisions. Although the clinical evaluation remains central for the diagnosis of DOC patients, several neurophysiological techniques and metrics (2) can help in stratifying patients and reduce misdiagnosis.

In this respect, the mini review by Sebastiano et al. highlights the EEG as the simplest neurophysiological method for assessing the severity of brain damage both in the acute and chronic stages of DOC, especially when used to compare DOC sub-populations or to longitudinally monitor the evolution of the brain function along with the clinical history of the patient. The limits and advantages of different quantitative methods to analyze the EEG signals including spectral, entropy, or coherence analyses are discussed in the review. The review by Pauli et al. focuses on resting-state EEG and points to the association between alpha power and better prognosis suggesting that alpha activity reflects intact thalamo-cortical loops but also the potential for directed attention in a recovering patient. This is in line with the recent International Federation of Clinical Neurophysiology (IFCN) (2) consensus indicating α-band power and advanced analysis of connectivity as relevant EEG markers to stratify and prognosticate DOC.

Reactivity to external stimuli, and evoked or event-related potentials (EP or ERP) can integrate EEG recordings adding valuable information about the integrity of sensory pathways and arousal systems. As an example of this approach, Perez et al. present an original study revealing the presence of an ERP Global Effect (ERP-GE; previously suggested as a correlate of conscious processing in the local-global auditory task) as a specific predictor of behavioral recovery of consciousness. All patients in a Minimally Conscious State+ (MCS+) or those who exited MCS (EMCS) with a significant ERP-GE recovered univocal overt behavioral evidence of consciousness. The authors found that the presence of a “global effect” can predict the recovery of the level of consciousness with a positive predictive value of 80% and a specificity of 84%, but with a very low sensitivity (35%). Thus, they could not observe any reliable prognostic value of ERP-GE in clinically unresponsive waking syndrome (UWS) or MCS- patients.

Increasing evidence suggests that 20% of behaviorally unresponsive DOC patients might show signs of residual consciousness using active neuroimaging or electrophysiological paradigms to reveal it. Cognitive–motor dissociation (CMD), as originally coined by Schiff (3), is the term used to identify these apparently unresponsive patients. Li et al. present a pilot study on functional near-infrared spectroscopy (fNIRS) using an active command-driven motor imagery paradigm to reveal covert consciousness, showing that fNIRS can be used to detect hemodynamic responses reflecting residual cognition in patients with prolonged DOC. Although preliminary and based on a small sample, their results are relevant given that fNIRS is a simple tool as compared to fMRI and can be potentially used at the bedside, especially in the acute phase after a brain injury, when the sensitivity of standard behavioral testing (essentially based on motor reactivity) is low and hampered by several confounding clinical factors potentially interfering with the production of behavioral or motor and verbal responses to external stimuli (4). The development of appropriate and feasible paraclinical testing is crucial to facilitate the discrimination of patients with CMD from those with DOC. The identification of potential CMD patients in an early phase is relevant since the prognostic trajectory appears to be different, with a better evolution over time in the majority of these patients (5, 6).

A second critical aspect of DOC management is the lack of therapeutical opportunities to integrate rehabilitation management. Pharmacological trials only revealed a mild efficacy of amantadine (7) therefore significant efforts have been focused on the possible restorative effect of electrophysiological modulation by means of deep brain stimulation, repetitive transcranial magnetic stimulation (rTMS), transcranial direct current stimulation (tDCS), and vagal nerve stimulation. The aim of such interventions is to modulate cortical excitability, stimulate arousability and functional integration (e.g., connectivity) within thalamo-cortical networks to facilitate the emergence of consciousness (8). In this vein, He et al. examined the therapeutic application of rTMS in DOC patients. rTMS is known to be able to modulate cortical excitability, either inducing a long-term potentiation or depression according to the frequency of stimulation. rTMS was able to induce a specific increase of the relative power of the alpha band. The finding converges with the mentioned evidence of a potential role of alpha activity as a marker of thalamocortical integrity. Another potential approach is tDCS that, according to the extensive review by Aloi et al., can lead to measurable neural changes in a subset of DOC patients. However, the high heterogeneity of stimulation parameters, montages, protocols, and outcome measures, do not yet allow us to draw any conclusions about the clinical applicability of tDCS. Rehabilitation of severely injured patients with DOC aims at stimulating recovery of sensory perception and interaction of patients with themselves and their environment. In this context, therapeutical approaches based on tDCS or rTMS are especially relevant since they might help the global recovery process by directly improving attention and arousability.

Overall, the original and review articles found in the present Research Topic on “*New Advances in Diagnostic Tools and Rehabilitation of Disorders of Consciousness in the Acute Phase*” illustrate that research in this field is close to identifying reliable tools to support the clinical assessment and prognosis of severely brain-injured patients. Hampering this process is the lack of homogeneity between different studied populations, the non-negligible risk of misdiagnosis of DOC (9), and the lack of objective read-outs of the effects of therapeutic methods. This special issue highlights recent advances in this vibrant field and prompts for extensive multicentric studies sharing common and accurate diagnostic classification.

## Author Contributions

All authors listed have made a substantial, direct and intellectual contribution to the work, and approved it for publication.

## Funding

This work was supported by Fondazione Regionale per la Ricerca Biomedica (Regione Lombardia), Project ERAPERMED2019-101, GA779282, by the Tiny Blue Dot Foundation (to MR).

## Conflict of Interest

The authors declare that the research was conducted in the absence of any commercial or financial relationships that could be construed as a potential conflict of interest.

## Publisher's Note

All claims expressed in this article are solely those of the authors and do not necessarily represent those of their affiliated organizations, or those of the publisher, the editors and the reviewers. Any product that may be evaluated in this article, or claim that may be made by its manufacturer, is not guaranteed or endorsed by the publisher.
